# Beyond symptoms: why do patients see the doctor?

**DOI:** 10.3399/bjgpopen20X101088

**Published:** 2020-05-20

**Authors:** André Hajek, Hans-Helmut König

**Affiliations:** 1 Department of Health Economics and Health Services Research, University Medical Center Hamburg-Eppendorf, Hamburg, Germany

**Keywords:** Doctor visits, General practitioner, Andersen model, Psychosocial, Personality, Primary health care, General practice, Locus of control, Preventive healthcare use

## Introduction

Why look at the determinants of doctor visits? Knowledge about the determinants of healthcare use is important to manage healthcare use, and to avoid its misuse as well as over- and under-use. To date, most studies examining the determinants of healthcare use refer to the widely-applied Andersen model. This model distinguishes between predisposing characteristics (such as sex or age), enabling resources (such as income or status of health insurance), and need factors (such as self-rated health or chronic conditions). A systematic review published in 2012 showed that need factors are important determining factors for individual healthcare service use.^1^ However, it appears plausible that other factors are also important, such as (1) personality characteristics (for example, neuroticism), or (2) psychosocial factors like loneliness (the state in which a person’s social network is smaller or less satisfying than desired), or life satisfaction (cognitive evaluation of life as a whole).

## Recent findings

Let’s take a closer look at some recent findings. Adjusting for various covariates included in the Andersen model, it was shown that personality factors, such as extraversion and neuroticism, are related to physician visit rates in the US and Germany.^2,3^ Extraverted individuals are, for example, talkative, outgoing, energetic, and sociable; therefore, extraverted individuals may have a more risk-prone (and injury-related) lifestyle. Furthermore, extraversion is associated with smoking and alcohol intake. A more speculative explanation may be that extraverted individuals may talk with their friends or acquaintances about their ailments, and their peer-group may encourage the extraverted individuals to visit the doctor. Neuroticism refers to the tendency to experience negative emotional states (including anxiety, depression, or anger). Factors such as negative emotions or perceived stress may lead to more doctor visits. In a 2017 study by the present authors, it was shown that the impact of self-rated health on physician visits differs depending on the level of neuroticism.^3^ For example, in those with bad self-rated health, neurotic individuals visit the doctor more often compared to less neurotic individuals, which intuitively appears to be plausible.

A further study has shown that external locus of control (the perception that outcomes are based on external factors) is important for physician visits.^4^ Individuals with high external locus of control may simply think that their health outcomes are based on visiting the doctor more frequently. In other words, patients with a high external locus of control may strongly believe in the fact that their GP can help them. On the other hand, those with a low external locus of control may simply not believe that GPs can treat their ailments.

Additionally, a nationally representative study recently showed that the number of GP visits increased with poorer subjective wellbeing and lower optimism.^5^ The link between subjective wellbeing and GP visits appears to be plausible because subjective wellbeing is strongly associated with somatic complaints. These complaints are linked to increased visits. Even other psychosocial factors, such as meaning in life, can play a role in physician visits.^6^

Similar findings were also seen for the determinants of frequent attendance.^7,8^ This is of particular importance because frequent attendance places a tremendous burden on the healthcare system. For example, a nationally representative study has shown that frequent GP visits are, among other things, associated with lower life satisfaction and self-esteem, and higher negative affect and perceived stress.^8^ In a 2018 study by Cruwys *et al*, a higher frequency of primary care attendance was shown to be associated with higher social isolation.^7^ Similar findings were made for preventive healthcare services.^9–11^ For example, a link between loneliness at baseline and a subsequent decreased use of the flu vaccine has been demonstrated.^12^

It is worth stressing that these are not spurious associations that are not generalisable: the findings cited above are based on nationally representative samples, performed sensitivity analyses, and are partly based on longitudinal study designs using regression models specifically designed for longitudinal data. Furthermore, these studies were typically adjusted for various covariates such as sex, age, income, self-rated health, physical illnesses, and depression in regression analysis.

## Conclusions

The authors would like to conclude their work with some general recommendations for future research:

1. This is a call for further interdisciplinary research when examining the determinants of healthcare use. This means, extending understanding of the determinants of healthcare use beyond those detailed in Andersen’s model, to include psychosocial factors and personality characteristics. For example, given demographic ageing and the geographical distance between family members, looking at the direct link between loneliness, social isolation, and doctor visits, particularly among older individuals, may be important. Does the frequency of physician visits change when older individuals lose their spouse, their best friends, or acquaintances?
2. Future research is required to clarify the role of psychosocial or personality factors that moderate the link between need factors and doctor visits. For example, while one individual with bad self-rated health may avoid visiting the GP, another individual with equally bad self-rated health may be a frequent attender simply because he or she scores high in neuroticism or external locus of control.
3. These recommendations are not restricted to general healthcare use or frequent GP visits. When analysing the determinants of preventive healthcare services, as already outlined, it is also important to broaden the research perspective. For example, the link between self-esteem and prostate cancer examination in men should be investigated in future research.
4. The link between psychosocial factors, personality characteristics, and doctor visits should be investigated in more countries (with different healthcare systems). For example, how are personality characteristics and GP visits associated in African or Asian countries?

The authors hope that the current work will inspire new research in this area and determine which other factors (beyond need) determine the frequency of doctor visits.
